# New Insights into the PPAR****γ**** Agonists for the Treatment of Diabetic Nephropathy

**DOI:** 10.1155/2014/818530

**Published:** 2014-01-29

**Authors:** Zhanjun Jia, Ying Sun, Guangrui Yang, Aihua Zhang, Songming Huang, Kristina Marie Heiney, Yue Zhang

**Affiliations:** ^1^Department of Nephrology, Nanjing Children's Hospital, Nanjing Medical University, Nanjing 210008, China; ^2^Institute of Pediatrics, Nanjing Medical University, Nanjing, China; ^3^Key Pediatric Laboratory of Nanjing City, Nanjing 210008, China; ^4^Institute for Translational Medicine and Therapeutics, University of Pennsylvania, Philadelphia, PA 19104, USA; ^5^Department of Internal Medicine, University of Utah, Salt Lake City, UT 84132, USA

## Abstract

Diabetic nephropathy (DN) is a severe complication of diabetes and serves as the leading cause of chronic renal failure. In the past decades, angiotensin-converting enzyme inhibitors (ACEIs)/angiotensin II receptor blockers (ARBs) based first-line therapy can slow but cannot stop the progression of DN, which urgently requests the innovation of therapeutic strategies. Thiazolidinediones (TZDs), the synthetic exogenous ligands of nuclear receptor peroxisome proliferator-activated receptor-**γ** (PPAR**γ**), had been thought to be a promising candidate for strengthening the therapy of DN. However, the severe adverse effects including fluid retention, cardiovascular complications, and bone loss greatly limited their use in clinic. Recently, numerous novel PPAR**γ** agonists involving the endogenous PPAR**γ** ligands and selective PPAR**γ** modulators (SPPARMs) are emerging as the promising candidates of the next generation of antidiabetic drugs instead of TZDs. Due to the higher selectivity of these novel PPAR**γ** agonists on the regulation of the antidiabetes-associated genes than that of the side effect-associated genes, they present fewer adverse effects than TZDs. The present review was undertaken to address the advancements and the therapeutic potential of these newly developed PPAR**γ** agonists in dealing with diabetic kidney disease. At the same time, the new insights into the therapeutic strategies of DN based on the PPAR**γ** agonists were fully addressed.

## 1. Introduction

PPARs are nuclear receptors consisting of three PPAR isoforms of PPAR*α*, PPAR*β*/*δ*, and PPAR*γ*. In the past decades, a number of studies demonstrated the critical role of PPARs in the regulation of metabolic homeostasis, inflammation, cell differentiation and proliferation, fluid balance, and so on [1–3]. Among three PPARs, PPAR*γ* was best characterized and its high-affinity ligands of TZDs were widely used in clinic for the treatment of type-2 diabetes mellitus (T2DM). PPAR*γ* is expressed in various organs with the most abundant expression in adipose tissue. It heterodimerizes with retinoid X receptor (RXR) and then binds to PPAR responsive element (PPRE) to regulate a number of target genes. TZDs including rosiglitazone, pioglitazone, and troglitazone are synthetic exogenous PPAR*γ* ligands with high efficacy in treating T2DM via enhancing the insulin sensitivity [3, 4]. Besides the potent role of TZDs in regulating hyperglycemia, they also effectively protect the kidneys from diabetic injury independently of its antihyperglycemia action [5–7]. Moreover, TZDs also displayed their capability of protecting the kidneys against other injuries beyond diabetes [8–11]. Although these beneficial effects of TZDs are so attractive and valuable, the severe side effects including fluid retention, cardiovascular complications, hepatotoxicity, and bone fractures greatly limited their use in clinic [12–14]. Interestingly, recent reports related to nitro-oleic acid, an endogenous PPAR*γ* ligand, demonstrated a potent renal-protective role under diabetic and nondiabetic situations possibly via PPAR*γ* dependent and independent mechanisms with no obvious side effects seen in TZDs [15–20]. More importantly, numerous selective PPAR*γ* agonists, also termed as selective PPAR*γ* modulators (SPPAR*γ*Ms), are being generated and some of them are under the clinical trials for the treatment of T2DM [12, 21]. The present review was undertaken to introduce and analyze the role of the exogenous and endogenous PPAR*γ* agonists and the SPPAR*γ*Ms in the protection of DN. Meanwhile, the therapeutic strategies via manipulating the use of various PPAR*γ* agonists will be fully addressed.

## 2. Role of PPAR*γ* in Diabetic Podocyte Injury and Proteinuria

With the profound increase of obesity, the prevalence of T2DM is rapidly rising worldwide. Among the patients with T2DM, about 10% of them developed DN [22]. In North America and Europe, DN serves as the leading cause of end-stage renal disease (ESRD). Proteinuria in DN patients is not only an established marker of DN progression, but it also plays a causative role in promoting inflammation and tubulointerstitial fibrosis. The occurrence of proteinuria in DN is due to the excessive passage of protein into the urine through the impaired glomerular filtration barrier (GFB) which is formed by endothelial cells, glomerular basement membrane (GBM), and podocytes. Accumulating evidence indicated the extreme importance of podocytopathy in diabetic glomerular damage [23]. The pathological manifestations of podocytopathy in DN include the cellular hypertrophy, foot process effacement, apoptosis, and detachment from the GBM [24, 25]. Glycemic control and pharmacological intervention using the ACEIs and/or ARBs only slow but cannot stop the DN progression. Therefore, to find more effective therapeutic strategies in countering the diabetes-associated renal injury is of vital importance and urgency.

PPAR*γ* is located in all three types of glomerular cells with a prominent expression in podocytes [26, 27]. Several studies including a recent meta-analysis showed that Ala12 variant of PPAR*γ*2 is significantly associated with a reduced risk of albuminuria among patients with type-2 diabetes [28]. These results highly suggested a functional role of PPAR*γ* in glomeruli, particularly in the podocytes. In agreement with this concept, numerous reports including a meta-analysis of 15 original clinical studies involving 2860 patients convincingly demonstrated the significant efficacy of rosiglitazone or pioglitazone on diabetic proteinuria [5].

In addition to the clinical evidence mentioned above, numerous basic studies performed in diabetic animals and in vitro cells also proved the beneficial action of PPAR*γ* in diabetic kidney disease [6, 7, 26, 27, 29]. Although the role of PPAR*γ* in treating diabetic kidney disease was extensively investigated since PPAR*γ* was discovered, the chief mechanism is roughly focused on the inhibition of inflammation and oxidative stress [8] with poorly understood molecular mechanisms.

A number of in vivo and in vitro studies demonstrated that PPAR*γ* benefits all kinds of kidney cells including the glomerular mesangial cells, endothelial cells, podocytes, and tubular epithelial cells under the diabetic condition [30] with more research emphasis on the podocytes [6, 7, 27, 31, 32]. The possible podocyte-protective mechanisms shown by literatures include the reversing of G1-phase cell circle [27], blockade of stretch-induced AT1 upregulation [7], and antiapoptosis effect [31, 32]. Recently, some reports elucidated the dysfunction of mitochondria in podocytes under the hyperglycemic status [33, 34]. It is known that dysfunctional mitochondria will generate excessive reactive oxygen species (ROS) and release the proapoptotic proteins, which subsequently leads to the cell and tissue damage. Thus, we can reasonably speculate that diabetes-associated mitochondria dysfunction in kidney, especially in podocytes, may contribute to the occurrence and the progression of DN. Moreover, Zhu et al. reported that PPAR*γ* activation remarkably improved the mitochondria dysfunction induced by aldosterone in podocytes [35]. These novel findings highly suggested that a mitochondria-protective effect may serve as an important mechanism of PPAR*γ* in opposing the diabetic podocyte injury. However, a direct link between the PPAR*γ* and mitochondria function in podocytes and other kidney cells under the diabetic condition does need a great deal of experimental evidence.

## 3. Limitations of TZDs in Treatment of DN

Although there is much evidence from clinical trials and basic studies pronounced the protective role of TZDs in DN, the severe side effects greatly restricted their use in patients. Troglitazone had to quit the market owing to the severe hepatotoxicity. Rosiglitazone has been found to be significantly associated with the increased risk of cardiovascular complications including heart failure and myocardial infarction leading to the restriction or withdrawal from the markets. As for the pioglitazone, it has been thought to have a different safety profile with no increase of cardiovascular disease as compared with other TZDs [36]. But, it still conserves the effects of bodyweight gain, bone loss, edema, and fluid retention which may increase the incidence of congestive heart failure [36]. Besides an established role of renal collecting duct PPAR*γ* in TZDs-induced fluid retention [37, 38], PPAR*γ* in the vasculature also played a crucial role in mediating the fluid retaining effect [39, 40]. All these findings delineated a mechanistic picture of PPAR*γ*-mediated fluid retention and also suggested some potential targets to overcome the TZD-induced fluid volume expansion. In addition to the fluid retaining effect, TZDs also cause the cardiomyocytes hypertrophy and coronary artery lesions with elusive mechanisms [41]. In general, TZD-induced cardiomyocytes hypertrophy was thought to possibly occur through the fluid retention-dependent and fluid retention-independent mechanisms [41]. Collectively, fluid retention and the detrimental effect of TZDs on the cardiomyocytes and cardiovascular system have to be avoided or minimized in the development of novel PPAR*γ* agonists or therapeutic strategies.

## 4. Strategies Based on Minimizing Adverse Effects of PPAR*γ* Agonists

### 4.1. Adjustment of the Therapeutic Dose of TZDs

Evidence from studies demonstrated the dose-dependent response of TZDs in antagonizing hyperglycemia of T2DM [42, 43]. Accordingly, the side effects including fluid retention and bodyweight gain were also promoted with the dose increasing [42, 43]. Theoretically, it is possible to optimize a lower dose of TZDs with significant protection of DN without severe adverse effects seen in higher dose of TZDs. Certainly this strategy may sacrifice some glucose-lowering efficacy of TZDs. In agreement with this notion, a low dose of rosiglitazone at 1 mg/kg/day for 7 weeks in STZ diabetic rats significantly lowered the proteinuria and attenuated pathological changes in glomeruli in parallel with reduced renal oxidative stress [44]. Unfortunately, the authors in this report did not show the evidence of TZD-related side effects such as bodyweight gain, Hct change, and plasma volume status [44]. To further validate this hypothesis, the extensive animal studies and clinical trials need to be performed in the future.

### 4.2. Endogenous PPAR*γ* Agonists Nitro-Oleic Acid for the Treatment of DN

Endogenous ligands for PPAR*γ* include unsaturated and oxidized fatty acids, nitrated fatty acids, eicosanoids, and prostaglandins [45]. 15-Deoxy-delta12, 14-prostaglandin J2 (15d-PGJ2), and nitro-oleic acid are well-recognized endogenous PPAR*γ* ligands and received attention from a number of studies [15–19, 46, 47]. Particularly, the effect of nitro-oleic acid on diabetes and diabetic kidney injury was evaluated [17, 47]. Infusion of nitro-oleic acid normalized the hyperglycemia in a type-2 diabetic model of db/db mice without affecting the bodyweight, an important indicator of fluid retention and fat accumulation [47]. A separate study from our group also found that nitro-oleic acid significantly attenuated proteinuria and metabolic syndrome in diabetic Zucker rats without affecting Hct, a widely used index of fluid retention in TZD models [19]. Most recently, our group gave evidence that nitro-oleic acid in combination with losartan, one of the ARBs, significantly ameliorated proteinuria and podocyte injury in diabetic db/db mice possibly via suppressing oxidative stress and inflammation [17]. In contrast, losartan alone failed to display the therapeutic efficacy during two weeks of treatment. All these results highly suggested that endogenous PPAR*γ* agonists may play a similar role as TZDs in protecting DN with no significant side effects shown by TZDs. Although nitro-oleic acid definitely activates PPAR*γ* [47], the detailed mechanism related to the beneficial role of nitro-oleic acid in opposing the diabetic kidney injury remains uncertain due to its nonselective activation of PPAR [48]. Furthermore, additional animal studies and clinical trials are needed to fully evaluate the safety and efficacy of nitro-oleic acid in treating DN, as well as hyperglycemia.

### 4.3. Selective PPAR*γ* Modulators for the Treatment of DN

TZDs, as full PPAR*γ* agonists, nonselectively regulate the expressions of antidiabetic efficacy-associated and adverse effect-associated genes in similar proportion [49], which leads to the overlap of dose response curves for therapeutic effect and side effect. Therefore, selective PPAR*γ* modulators (SPPAR*γ*Ms) are being actively pursued as the second generation of PPAR*γ* agonists. Presumably, SPPAR*γ*Ms preserve greater capability in the regulation of antidiabetic genes than that of adverse-effect-associated genes, which could effectively limit the side effects seen in TZDs, particularly the fluid retention. By now, numerous synthetic SPPAR*γ*Ms have been generated [21]. Among them, balaglitazone is the prominent one and is currently under the phase III clinical trials in the United States and Europe [50]. Data from the clinical trials showed a robust antidiabetic effect of balaglitazone with less incidence of fluid retention and fat accumulation [50]. The preclinical data of this drug also indicated less fluid retention, less heart hypertrophy, and no signs of bone loss [50]. Besides balaglitazone, INT131 also reached human trials. Data from animals showed no significant fluid retention, bodyweight gain, cardiac hypertrophy, and bone loss with similar glucose-lowering effect as TZDs [49]. Human studies also showed that INT131 at doses from 0.5 to 3 mg per day effectively lowered blood glucose in patients with type-2 diabetes without causing edema [49]. Although these SPPAR*γ*Ms convincingly demonstrated the antihyperglycemia effect with fewer side effects, their efficacy in treating DN is still unclear. We believe, with better recognition on the importance of SPPAR*γ*Ms and the research progression of this field, this question will be answered soon.

## 5. Strategies Based on Increasing the Efficacy of PPAR*γ* Agonists

### 5.1. Combination of RAS Blockers with PPAR*γ* Agonists

RAS blockers including ACEIs and ARBs served as the cornerstone therapy of DN in the past decades. Although their efficacy in reducing the proteinuria and retarding the DN progression was established, a large number of DN patients with the therapy of RAS blockade and glycemic control still stepped onto the stage of renal failure. This situation raised a serious request for more effective therapies of DN. Due to the established role of PPAR*γ* in protecting DN, TZDs partnering with ACEI and/or ARB served to be a better option for the nephrologists. However, the unacceptable side effects of TZDs unfortunately interrupted such an ideal marriage. Even so, we still believe that with the discovery and the clinical application of novel PPAR*γ* agonists including endogenous PPAR*γ* agonists and SPPAR*γ*Ms, this marriage between RAS blocker and PPAR*γ* agonist will be rebuilt in the near future. Currently, an exciting example is telmisartan with dual properties of AT1 blocker and selective PPAR*γ* modulator [51, 52]. But, it still needs the evidence from clinical trials and basic studies to certify that telmisartan could play a better role than a specific AT1 blocker alone for the treatment of DN. Moreover, a combination of low dose TZDs with RAS blockers is also worth consideration.

### 5.2. Dual Activation of PPAR*γ* and PPAR*α*

PPAR*α* is distributed in several tissues including the kidney and its agonists had shown the pivotal roles in regulating lipid metabolism, inflammation, and cardiovascular response [53]. Recent reports demonstrated that PPAR*α* agonists such as fenofibrate can effectively protect DN via reducing renal lipotoxicity and inhibiting renal inflammation and oxidative stress [54]. These findings highly suggested that PPAR*α* may serve as a new therapeutic target of DN. In addition, dual activation of PPAR*α* and PPAR*γ* may be a novel strategy against DN. In line with this concept, an animal study using combined low dose PPAR*α* agonist fenofibrate and low dose PPAR*γ* agonist rosiglitazone more remarkably attenuated the diabetic kidney injury than drug alone [44]. Moreover, the dual PPAR*α*/*γ* agonist tesaglitazar markedly ameliorated the diabetic renal injury in db/db mice [55] and obese Zucker rats [56]. Based on this notion and some research findings, we can conceive that a combination of PPAR*α* agonist with novel PPAR*γ* agonists or low dose of TZDs could be a suitable strategy for the treatment of DN.

### 5.3. Blockade of COX-2/PGE2/EP Pathway Partnering with PPAR*γ* Agonists

COX-2 was induced in the podocytes under the diabetic status [57]. Inhibition of COX-2 or interruption of PGE2 receptors EP1/EP4 significantly attenuated diabetic kidney injury [58–61]. Under some nondiabetic conditions, such as the chronic kidney disease model of 5/6 nephrectomy and acute kidney injury model of adriamycin nephropathy, COX-2/PGE2/EP4 pathway also played a detrimental role in podocytes [62]. These results highly suggested a new therapeutic target of COX-2/PGE2/EP pathway in treating DN. Unfortunately, the increased cardiovascular mortality and morbidity and the fluid retaining effect of the COX inhibitors limited their long-term application in clinic [63]. In theory, blockade of COX-2/PGE2/EP pathway in combination with PPAR*γ* agonists will cause greater extracellular fluid volume expansion and more severe cardiovascular complications. However, low dose aspirin has been used for long-term primary or secondary prevention of vascular disease in clinic and the safety has been well evaluated. Although there is no convincing evidence showing the efficacy of low dose COX inhibitors in the therapy of DN, the combination of a lower dose COX inhibitor with SPPAR*γ*M or endogenous PPAR*γ* agonist could be a feasible strategy in treating DN. Moreover, it is also worthwhile to investigate whether a low dose of COX inhibitor will strengthen the effect of RAS inhibitors in protecting the diabetic kidney. By reviewing the literatures, we did not find any clinical or animal reports demonstrating this notion.

mPGES-1 is one of three characterized prostaglandin E synthases (mPGES-1, mPGES-2, and cPGES). In the past decade, only mPGES-1 was evidenced as a functional PGE2 synthase in vivo and played important roles under various physiological and pathological conditions [64–71]. Evidence from mPGES-2 and cPGES KO mice strongly argued against their property of PGE2 synthesis [72, 73]. mPGES-1 mediated the injury in some kidney injury models [71, 74]. For example, in a 5/6 nephrectomy mouse model, mPGES-1 deletion significantly reduced proteinuria and attenuated glomerular injury and podocyte damage possibly through the inhibition of inflammation and oxidative stress [71]. However, in a STZ diabetic mouse model, renal mPGES-1 was not regulated by hyperglycemia and deletion of mPGES-1 did not affect renal PGE2 production and glomerular injury. This largely excluded the involvement of mPGES-1 in mediating the renal PGE2 induction and kidney injury in type-1 diabetes, at least in mouse (unpublished data). Oppositely, in a type-2 diabetic model of db/db mouse, mPGES-1 was remarkably elevated in the glomeruli [75]. This discrepancy of mPGES-1 regulation may reflect the difference of the pathogenic mechanism and disease status of DN between the type-1 and type-2 diabetes. More interestingly, one-week rosiglitazone treatment abolished mPGES-1 induction in glomeruli without affecting COX-2 expression in these db/db mice. This result suggested that inhibition of COX-2 in combination with PPAR*γ* agonist may provide additional protection from diabetic kidney disease. In addition, it is also expected that antagonism of specific PGE2 receptors partnering with a selective PPAR*γ* agonist could achieve better outcome in DN treatment than PPAR*γ* agonist alone. However, none of the mPGES-1 inhibitors or EP antagonists is available in clinic now. The investigations in animals or in vitro cells may be the current emphasis to validate the present hypothesis.

## 6. Perspectives

Except for the known side effects, TZDs have been so fantastic for the treatment of T2DM and diabetic kidney disease. We believe that the withdrawal or restriction of TZDs owing to their severe side effects only temporarily fades the light of PPAR*γ* in treating human diseases. With the development of novel PPAR*γ* agonists with minimal side effects, the PPAR*γ* will gain the researcher's focus again. Actually, PPAR*γ* activation not only ameliorates diabetic kidney disease, but it also protects kidneys from a variety of other acute and chronic insults. In the past decades, only RAS blockers stand on the first line in fighting against chronic kidney diseases (CKDs). With the generation and application of novel PPAR*γ* agonists in the near future, we can conceive that the therapeutic outcome of DN and other CKDs will be significantly advanced.

## References

[1] Evans RM, Barish GD, Wang Y-X (2004). PPARs and the complex journey to obesity. *Nature Medicine*.

[2] Barish GD, Narkar VA, Evans RM (2006). PPAR*δ*: a dagger in the heart of the metabolic syndrome. *The Journal of Clinical Investigation*.

[3] Poulsen L, Siersbæk M, Mandrup S (2012). PPARs: fatty acid sensors controlling metabolism. *Seminars in Cell & Developmental Biology*.

[4] Tontonoz P, Spiegelman BM (2008). Fat and beyond: the diverse biology of PPAR*γ*. *Annual Review of Biochemistry*.

[5] Sarafidis PA, Stafylas PC, Georgianos PI, Saratzis AN, Lasaridis AN (2010). Effect of thiazolidinediones on albuminuria and proteinuria in diabetes: a meta-analysis. *American Journal of Kidney Diseases*.

[6] Yang H-C, Ma L-J, Ma J, Fogo AB (2006). Peroxisome proliferator-activated receptor-gamma agonist is protective in podocyte injury-associated sclerosis. *Kidney International*.

[7] Miceli I, Burt D, Tarabra E, Camussi G, Perin PC, Gruden G (2010). Stretch reduces nephrin expression via an angiotensin II-AT_1_-dependent mechanism in human podocytes: effect of rosiglitazone. *American Journal of Physiology*.

[8] Yang J, Zhou Y, Guan Y (2012). PPAR*γ* as a therapeutic target in diabetic nephropathy and other renal diseases. *Current Opinion in Nephrology and Hypertension*.

[9] Lepenies J, Hewison M, Stewart PM, Quinkler M (2010). Renal PPAR*γ* mRNA expression increases with impairment of renal function in patients with chronic kidney disease. *Nephrology*.

[10] Sugawara A, Uruno A, Kudo M, Matsuda K, Yang CW, Ito S (2010). Effects of PPAR*γ* on hypertension, atherosclerosis, and chronic kidney disease. *Endocrine Journal*.

[11] Fogo AB (2011). PPAR*γ* and chronic kidney disease. *Pediatric Nephrology*.

[12] Ahmadian M, Suh JM, Hah N (2013). PPAR*γ* signaling and metabolism: the good, the bad and the future. *Nature Medicine*.

[13] Kung J, Henry RR (2012). Thiazolidinedione safety. *Expert Opinion on Drug Safety*.

[14] Nissen SE, Wolski K (2007). Effect of rosiglitazone on the risk of myocardial infarction and death from cardiovascular causes. *The New England Journal of Medicine*.

[15] Zhang J, Villacorta L, Chang L (2010). Nitro-oleic acid inhibits angiotensin II-induced hypertension. *Circulation Research*.

[16] Wang H, Liu H, Jia Z (2010). Nitro-oleic acid protects against endotoxin-induced endotoxemia and multiorgan injury in mice. *American Journal of Physiology*.

[17] Liu Y, Jia Z, Liu S (2013). Combined losartan and nitro-oleic acid remarkably improves diabetic nephropathy in mice. *American Journal of Physiology*.

[18] Liu H, Jia Z, Soodvilai S (2008). Nitro-oleic acid protects the mouse kidney from ischemia and reperfusion injury. *American Journal of Physiology*.

[19] Wang H, Liu H, Jia Z, Guan G, Yang T (2010). Effects of endogenous PPAR agonist nitro-oleic acid on metabolic syndrome in obese zucker rats. *PPAR Research*.

[20] Kansanen E, Jyrkkänen H-K, Volger OL (2009). Nrf2-dependent and -independent responses to nitro-fatty acids in human endothelial cells: identification of heat shock response as the major pathway activated by nitro-oleic acid. *The Journal of Biological Chemistry*.

[21] Doshi LS, Brahma MK, Bahirat UA, Dixit AV, Nemmani KVS (2010). Discovery and development of selective PPAR*γ* modulators as safe and effective antidiabetic agents. *Expert Opinion on Investigational Drugs*.

[22] C. Centers for Disease, Prevention (2005). Incidence of end-stage renal disease among persons with diabetes—United States, 1990–2002. *Morbidity and Mortality Weekly Report*.

[23] Weil EJ, Lemley KV, Mason CC (2012). Podocyte detachment and reduced glomerular capillary endothelial fenestration promote kidney disease in type 2 diabetic nephropathy. *Kidney International*.

[24] Susztak K, Raff AC, Schiffer M, Böttinger EP (2006). Glucose-induced reactive oxygen species cause apoptosis of podocytes and podocyte depletion at the onset of diabetic nephropathy. *Diabetes*.

[25] Diez-Sampedro A, Lenz O, Fornoni A (2011). Podocytopathy in diabetes: a metabolic and endocrine disorder. *American Journal of Kidney Diseases*.

[26] Guan Y, Breyer MD (2001). Peroxisome proliferator-activated receptors (PPARs): novel therapeutic targets in renal disease. *Kidney International*.

[27] Okada T, Wada J, Hida K (2006). Thiazolidinediones ameliorate diabetic nephropathy via cell cycle-dependent mechanisms. *Diabetes*.

[28] de Cosmo S, Prudente S, Lamacchia O (2011). PPAR*γ*2 P12A polymorphism and albuminuria in patients with type 2 diabetes: a meta-analysis of case-control studies. *Nephrology Dialysis Transplantation*.

[29] Liang YJ, Jian JH, Chen CY, Hsu CY, Shih CY, Leu JG (2013). L-165,041, troglitazone and their combination treatment to attenuate high glucose-induced receptor for advanced glycation end products (RAGE) expression. *European Journal of Pharmacology*.

[30] Qian Y, Feldman E, Pennathur S, Kretzler M, Brosius FC (2008). From fibrosis to sclerosis: mechanisms of glomerulosclerosis in diabetic nephropathy. *Diabetes*.

[31] Kanjanabuch T, Ma L-J, Chen J (2007). PPAR-*γ* agonist protects podocytes from injury. *Kidney International*.

[32] Miglio G, Rosa AC, Rattazzi L (2011). The subtypes of peroxisome proliferator-activated receptors expressed by human podocytes and their role in decreasing podocyte injury. *British Journal of Pharmacology*.

[33] Wang W, Wang Y, Long J (2012). Mitochondrial fission triggered by hyperglycemia is mediated by ROCK1 activation in podocytes and endothelial cells. *Cell Metabolism*.

[34] Stieger N, Worthmann K, Teng B (2012). Impact of high glucose and transforming growth factor-*β* on bioenergetic profiles in podocytes. *Metabolism*.

[35] Zhu C, Huang S, Yuan Y (2011). Mitochondrial dysfunction mediates aldosterone-induced podocyte damage: a therapeutic target of PPAR*γ*. *The American Journal of Pathology*.

[36] Shah P, Mudaliar S (2010). Pioglitazone: side effect and safety profile. *Expert Opinion on Drug Safety*.

[37] Zhang H, Zhang A, Kohan DE, Nelson RD, Gonzalez FJ, Yang T (2005). Collecting duct-specific deletion of peroxisome proliferator-activated receptor *γ* blocks thiazolidinedione-induced fluid retention. *Proceedings of the National Academy of Sciences of the United States of America*.

[38] Guan Y, Hao C, Cha DR (2005). Thiazolidinediones expand body fluid volume through PPAR*γ* stimulation of ENaC-mediated renal salt absorption. *Nature Medicine*.

[39] Sotiropoulos KB, Clermont A, Yasuda Y (2006). Adipose-specific effect of rosiglitazone on vascular permeability and protein kinase C activation: novel mechanism for PPAR*γ* agonist’s effects on edema and weight gain. *The FASEB Journal*.

[40] Yang T, Soodvilai S (2008). Renal and vascular mechanisms of thiazolidinedione-induced fluid retention. *PPAR Research*.

[41] Robinson E, Grieve DJ (2009). Significance of peroxisome proliferator-activated receptors in the cardiovascular system in health and disease. *Pharmacology & Therapeutics*.

[42] Aronoff S, Rosenblatt S, Braithwaite S, Egan JW, Mathisen AL, Schneider RL (2000). Pioglitazone hydrochloride monotherapy improves glycemic control in the treatment of patients with type 2 diabetes: a 6-month randomized placebo-controlled dose-response study. *Diabetes Care*.

[43] Nolan JJ, Jones NP, Patwardhan R, Deacon LF (2000). Rosiglitazone taken once daily provides effective glycaemic control in patients with type 2 diabetes mellitus. *Diabetic Medicine*.

[44] Arora MK, Reddy K, Balakumar P (2010). The low dose combination of fenofibrate and rosiglitazone halts the progression of diabetes-induced experimental nephropathy. *European Journal of Pharmacology*.

[45] Cariou B, Charbonnel B, Staels B (2012). Thiazolidinediones and PPAR*γ* agonists: time for a reassessment. *Trends in Endocrinology & Metabolism*.

[46] Ueki S, Kato H, Kobayashi Y (2007). Anti- and proinflammatory effects of 15-deoxy-Δ^12,14^-prostaglandin J_2_(15d-PGJ_2_) on human eosinophil functions. *International Archives of Allergy and Immunology*.

[47] Schopfer FJ, Cole MP, Groeger AL (2010). Covalent peroxisome proliferator-activated receptor *γ* adduction by nitro-fatty acids: selective ligand activity and anti-diabetic signaling actions. *The Journal of Biological Chemistry*.

[48] Menon MC, Chuang PY, He JC (2013). Nitro-oleic acid is a novel anti-oxidative therapy for diabetic kidney disease. *American Journal of Physiology*.

[49] Yew T, Toh S-A, Millar JS (2012). Selective peroxisome proliferator-activated receptor-*γ* modulation to reduce cardiovascular risk in patients with insulin resistance. *Recent Patents on Cardiovascular Drug Discovery*.

[50] Agrawal R, Jain P, Dikshit SN (2012). Balaglitazone: a second generation peroxisome proliferator-activated receptor (PPAR) gamma (*γ*) agonist. *Mini-Reviews in Medicinal Chemistry*.

[51] Lamotte Y, Martres P, Faucher N (2010). Synthesis and biological activities of novel indole derivatives as potent and selective PPAR*γ* modulators. *Bioorganic & Medicinal Chemistry Letters*.

[52] Dropinski JF, Akiyama T, Einstein M (2005). Synthesis and biological activities of novel aryl indole-2-carboxylic acid analogs as PPAR*γ* partial agonists. *Bioorganic & Medicinal Chemistry Letters*.

[53] Shah A, Rader DJ, Millar JS (2010). The effect of PPAR-*α* agonism on apolipoprotein metabolism in humans. *Atherosclerosis*.

[54] Balakumar P, Kadian S, Mahadevan N (2012). Are PPAR alpha agonists a rational therapeutic strategy for preventing abnormalities of the diabetic kidney?. *Pharmacological Research*.

[55] Cha DR, Zhang X, Zhang Y (2007). Peroxisome proliferator-activated receptor *α*/*γ* dual agonist tesaglitazar attenuates diabetic nephropathy in db/db mice. *Diabetes*.

[56] Liao J, Soltani Z, Ebenezer P (2010). Tesaglitazar, a dual peroxisome proliferator- activated receptor agonist (PPAR*α*/*γ*), improves metabolic abnormalities and reduces renal injury in obese zucker rats. *Nephron*.

[57] Stitt-Cavanagh E, MacLeod L, Kennedy C (2009). The podocyte in diabetic kidney disease. *The Scientific World Journal*.

[58] Cheng H-F, Wang CJ, Moeckel GW, Zhang M-Z, McKanna JA, Harris RC (2002). Cyclooxygenase-2 inhibitor blocks expression of mediators of renal injury in a model of diabetes and hypertension. *Kidney International*.

[59] Makino H, Tanaka I, Mukoyama M (2002). Prevention of diabetic nephropathy in rats by prostaglandin E receptor EP1-selective antagonist. *Journal of the American Society of Nephrology*.

[60] Mohamed R, Jayakumar C, Ramesh G (2013). Chronic administration of EP4-selective agonist exacerbates albuminuria and fibrosis of the kidney in streptozotocin-induced diabetic mice through IL-6. *Laboratory Investigation*.

[61] Cheng H, Fan X, Moeckel GW, Harris RC (2011). Podocyte COX-2 exacerbates diabetic nephropathy by increasing podocyte (pro)renin receptor expression. *Journal of the American Society of Nephrology*.

[62] Stitt-Cavanagh EM, Faour WH, Takami K (2010). A maladaptive role for EP4 receptors in podocytes. *Journal of the American Society of Nephrology*.

[63] Rios A, Vargas-Robles H, Gámez-Méndez AM, Escalante B (2011). Cyclooxygenase-2 and kidney failure. *Prostaglandins & Other Lipid Mediators*.

[64] Jia Z, Liu G, Sun Y (2013). mPGES-1-derived PGE_2_ mediates dehydration natriuresis. *American Journal of Physiology*.

[65] Jia Z, Liu G, Downton M, Dong Z, Zhang A, Yang T (2012). mPGES-1 deletion potentiates urine concentrating capability after water deprivation. *American Journal of Physiology*.

[66] Jia Z, Aoyagi T, Kohan DE, Yang T (2010). mPGES-1 deletion impairs aldosterone escape and enhances sodium appetite. *American Journal of Physiology*.

[67] Jia Z, Aoyagi T, Yang T (2010). mPGES-1 protects against DOCA-salt hypertension via inhibition of oxidative stress or stimulation of NO/cGMP. *Hypertension*.

[68] Jia Z, Wang H, Yang T (2009). Mice lacking mPGES-1 are resistant to lithium-induced polyuria. *American Journal of Physiology*.

[69] Soodvilai S, Jia Z, Wang M-H, Dong Z, Yang T (2009). mPGES-1 deletion impairs diuretic response to acute water loading. *American Journal of Physiology*.

[70] Soodvilai S, Jia Z, Yang T (2007). Hydrogen peroxide stimulates chloride secretion in primary inner medullary collecting duct cells via mPGES-1-derived PGE_2_. *American Journal of Physiology*.

[71] Jia Z, Wang H, Yang T (2012). Microsomal prostaglandin e synthase 1 deletion retards renal disease progression but exacerbates anemia in mice with renal mass reduction. *Hypertension*.

[72] Jania LA, Chandrasekharan S, Backlund MG (2009). Microsomal prostaglandin E synthase-2 is not essential for *in vivo* prostaglandin E_2_ biosynthesis. *Prostaglandins & Other Lipid Mediators*.

[73] Lovgren AK, Kovarova M, Koller BH (2007). cPGES/p23 is required for glucocorticoid receptor function and embryonic growth but not prostaglandin E_2_ synthesis. *Molecular and Cellular Biology*.

[74] Jia Z, Wang N, Aoyagi T, Wang H, Liu H, Yang T (2011). Amelioration of cisplatin nephrotoxicity by genetic or pharmacologic blockade of prostaglandin synthesis. *Kidney International*.

[75] Sun Y, Jia Z, Liu G (2013). PPAR*γ* agonist rosiglitazone suppresses renal mPGES-1/PGE2 pathway in db/db mice. *PPAR Research*.

